# On the Nature and Treatment of Apoplexy

**Published:** 1813-09

**Authors:** C. Abel

**Affiliations:** Halesworth


*To the Editors of the Medical and Physical Journal.*

GENTLEMEN,

AS Portal's valuable book on Apoplexy may not be in the possession of many of your readers, the following compressed translation of his two memoirs prefixed to that work, will perhaps be thought worthy a place in your Journal, particularly as they contain the substance of the doctrine expanded in the body of his publication.

*Halesworth*, I am, Gentlemen, your's, &c.

*August* 6, 1813. C. ABEL.

### *On the* NATURE *and* TREATMENT *of* APOPLEXY.

### *Memoir the First, by* PORTAL.

THE most ancient physicians considering Apoplexy as the effect of a compression of the brain, almost all recommended the same treatment, to which blood-letting was essential; but in later times physicians having divided this disease into two species, the sanguineous and serous or pituitous, imagined that each was announced by particular signs, and required a different treatment.

In sanguineous apoplexy, they remark, the countenance is more or less red, the eyes are prominent and shining, the pulse is full, and the veins of the face and neck appear gorged with blood.

In the serous or pituitous apoplexy, they add, the face is pale and livid, the mouth filled with foam, and the pulse more small and contracted than in the sanguineous. It is so much the more essential, says Sennert, with most of the physicians followers of his doctrine, to know the signs which distinguish serous from sanguineous apoplexy, as it is necessary to treat the two diseases differently; the remedies which are useful in the latter would be fatal in the former, particularly bleeding; it is the most powerful resource in sanguineous, and would be the most fatal in serous apoplexy. Such was the doctrine of the celebrated physicians who preceded us, and such is that of the most distinguished among the moderns. "A venæ sectione," says M. Lieutaud, "nimirum abstinere præstat; quæ tanto est nociva in hacce apoplexiæ specie, quanto propina in alterâ."

I had adopted this doctrine in my practice and in my lectures, when I had occasion to open the body of a barrister, who died after experiencing all the symptoms of serous apoplexy, as profound sleep, stertorous respiration, foaming at the mouth, and cadaverous paleness of the countenance; bleeding was not tried, an emetic and the volatile alkalies were administered, and blisters applied to the legs and nape of the neck.

It may be said that no remedy was omitted which had been prescribed in parallel cases by the masters of the art; yet these means were unavailing, though so strikingly indicated. Scarcely was this barrister dead, when the paleness of his face diminished, and it became in the space of two or three hours of a crimson redness, and the heat of the body more sensible than in the last moments of his life; and was so considerable twenty-four hours after death, that I thought it right to defer the opening of the body to the next day; but I made some scarifications in the bottom of his feet, and obtained about two spoonfuls of very red and liquid blood.

Forty hours after death the body was examined; it had now no warmth, and the visage was rather purple than pale. I opened the head with caution, and observed the following appearances: the vessels which wind along the pericranium, those of the dura and those of the pia mater, were full of blood; the vessels which meet between the circumvolutions of the brain, and in the anfractuosities of that viscus, were dilated and puffed up by this fluid; it appeared as if the brain was covered by an injected vascular lacework; the plexus choroides was equally gorged with blood, and there was much affused in the base of the cranium; the ventricles of the brain were dry, we found not a drop of affused water. These circumstances evidently proved to us that this case was one of sanguineous not of serous apoplexy; and that other remedies than those resorted to should have been tried, especially blood-letting.

The following is another example, proving that paleness of the countenance, foaming at the mouth, and contraction of the pulse, joined to coma and stertorous respiration, do not in any manner indicate serous apoplexy. In the month of June, 1773, M. Bertrand, brigadier of the grey musketeers, commanded a detachment of his company on the plain Des Sablons: his horse fell backwards upon him, and he could not rise; his countenance was of a cadaverous paleness, his pulse small and contracted, and his respiration became very oppressed and stertorous. This officer was supposed to have suffered an apoplexy of the humors, and in consequence a powerful emetic was administered without effect; and, what will scarcely be believed, he was not bled. Called to his assistance, I blooded him in the jugular; the pulse rose, and became more strong and regular; his senses appeared returning, he vomited a little, and moved his superior extremities. In the evening M. Borden was called in consultation; we applied blisters to the legs and nape of the neck; neck; vain resource! the patient relapsed into a stupor, and died with all the symptoms of apoplexy. I attended on the following day at the opening of the body with several physicians and surgeons; much blood was found in the cavity of the cranium affused under the hemispheres of the cerebrum and cerebellum, and the vertebral canal was full of concrete blood; the ventricles contained the usual quantity of serosity, which is more abundant as the opening of the body is longer delayed. This dissection satisfied us that bleeding should have been insisted on sooner, and to a greater extent. I could cite in this place other observations, the result of which would be the same.

Instructed by these errors, I bled in the foot and in the jugular some persons who were supposed to suffer under serous apoplexy, and by this means only they were preserved. The Marquis of Breda, two years ago, was attacked with apoplexy; he is a very large and coarse man, and was then about fifty-five years of age. He was found senseless in bed, with stertorous respiration; his face of a deadly hue, his lips covered with foam, his pulse small and contracted. From these symptoms he was thought to labor under serous apoplexy, and an emetic was prescribed, which had not operated when I arrived, and I advised an abundant bleeding from the foot. Whilst the blood flowed the pulse rose; the respiration, which was interrupted, short, and oppressed, became more free, but remained stertorous. We gave an emetic, which produced no effect. I recommended a second bleeding in the foot, which was scarcely finished when the patient moved his eyes, raised his eyelids, and appeared to consider the objects before him. We observed the lower lip to move at different times; these motions often precede vomiting, which very soon followed; the patient brought up a large quantity of frothy matter, and very little else; we gave him an emetic, and it produced a copious evacuation; the limbs recovered by degrees sensibility and motion; respiration almost regained its natural state, but he remained for several hours unconscious of the loudest sounds, and still longer without the power of speech. He was in this last state when I returned to him; he made several signs to render himself understood, which I could not comprehend, but I at length discovered that he wished to write, and he wrote with a trembling hand these words: "Do you not observe that I cannot speak." I advised a third bleeding, which, though not performed till two hours afterwards, was so successful that the patient spoke during the operation. The patient owed his re-establishment to the large bleedings: the blood accumulated in the vessels of the brain produced without doubt a compression of that organ, and of the origin of the nerves, which could no longer transmit sensibility to the viscera or mobility to the muscles. Was the emetic of no effect, and in what way could it act? It could only exercise its action by stimulating the stomach, which to disembarrass itself contracts in proportion to the sensibility of its nerves and the irritability of its muscular fibres. But as the stomach of the patient in question was as insensible as every other part, the emetic could not be effective; it is when the compression of the brain and nerves is diminished that the stomach regains a part of its sensibility, and becomes capable of receiving the impression of the emetic. In the same manner other organs gradually recovered their functions. The Marquis of Breda has since enjoyed good health.

I could here mention other very analogous observations, proving that the symptoms from which it has been usual to infer the existence of serous apoplexy are delusive, and that those who were supposed, in consequence of these symptoms, to suffer under serous, were affected with sanguineous apoplexy.

But if paleness of the countenance, foaming at the mouth, small and contracted pulse, joined to the other symptoms of apoplexy, do not surely indicate the presence of water in the cranium nor in the brain, redness of the face and fulness of the pulse are not more certain signs of excess of blood in those parts. Those who suffer under hydrocephalus, as is generally known, have the cheeks very red; but what is not equally so, is, that in many apoplectics who have had the countenance very red and the pulse very full, and who have not had foaming at the mouth, water has been found between the brain and cavity of the cranium, in the ventricles of the brain, or in both these situations at the same time.

The body of a man was brought into my private theatre whose face was tumefied, and of a black color, as if covered with echymosis. I thought this man had died of apoplexy produced by the stagnation of blood in the brain; but I was convinced to the contrary on opening the body. I found the ventricles of the brain full of a yellowish humor, and the plexus choroides covered with hydatids, two or three of which were as large as the seed of a grape, and full of water; others were torn, and perhaps had allowed the water to escape which the ventricles contained: however this might be, there was no blood stagnant in the vessels of the brain, or effused into the cavities of that viscus, or into those of the cranium.

In 1767, a butcher died with all the symptoms of sanguineous apoplexy; he was naturally very fat, and during the the attack was rather black than red: he had some foaming at the mouth, and his pulse was full and contracted. This patient died unrelieved by all the remedies which were promptlyadministered.

I assisted at the opening of the body by M. Leduc, and the following appearances were observed:—the ventricles of the brain were filled with a reddish serosity, and the plexus choroides were loaded with hydatids of a considerable volume.

We find in authors, and particularly in Morgagni and Lieutaud, some observations of a similar tendency with those just given; but as they have not drawn from them the same consequences which we have deduced, and as points of doctrine are involved in this discussion which cannot be too well established, whether we consider their importance or how little they are understood, I have thought it proper to bring into this memoir my own particular observations.

Anatomy is never more useful to medicine than when it unveils its errors.

I propose in another memoir to prove that the vessels of the brain are almost always gorged with blood when any serosity is affused into the tissue or cavities of that viscus; that serous is almost always the termination of sanguineous apoplexy; and that serous apoplexy without congestion of blood in the brain is a very rare occurrence.

*End of Memoir the First.*

P. S.—In my communication to Dr. Kinglake, No. 172, p. 454, the following sentence, "to every one who, by reasoning *or* the statement of facts," was mis-printed "*on* the statement of facts."

(To be continued.)

